# Bracken: estimating species abundance in metagenomics data

**DOI:** 10.7717/peerj-cs.104

**Published:** 2017-01-02

**Authors:** Jennifer Lu, Florian P. Breitwieser, Peter Thielen, Steven L. Salzberg

**Affiliations:** 1 Department of Biomedical Engineering, Johns Hopkins University, Baltimore, MD, United States; 2 Center for Computational Biology, McKusick-Nathans Institute of Genetic Medicine, Johns Hopkins School of Medicine, Baltimore, MD, United States; 3 Applied Physics Laboratory, Johns Hopkins University, Laurel, MD, United States; 4 Departments of Computer Science and Biostatistics, Johns Hopkins University, Baltimore, MD, United States

**Keywords:** Metagenomics, Species abundance, Microbiome, Bayesian estimation, Bioinformatics, Computational Biology

## Abstract

Metagenomic experiments attempt to characterize microbial communities using high-throughput DNA sequencing. Identification of the microorganisms in a sample provides information about the genetic profile, population structure, and role of microorganisms within an environment. Until recently, most metagenomics studies focused on high-level characterization at the level of phyla, or alternatively sequenced the 16S ribosomal RNA gene that is present in bacterial species. As the cost of sequencing has fallen, though, metagenomics experiments have increasingly used unbiased shotgun sequencing to capture all the organisms in a sample. This approach requires a method for estimating abundance directly from the raw read data. Here we describe a fast, accurate new method that computes the abundance at the species level using the reads collected in a metagenomics experiment. Bracken (Bayesian Reestimation of Abundance after Classification with KrakEN) uses the taxonomic assignments made by Kraken, a very fast read-level classifier, along with information about the genomes themselves to estimate abundance at the species level, the genus level, or above. We demonstrate that Bracken can produce accurate species- and genus-level abundance estimates even when a sample contains multiple near-identical species.

## INTRODUCTION

Metagenomics is a rapidly growing field of study, driven in part by our ability to generate enormous amounts of DNA sequence rapidly and inexpensively. Since the human genome was first published in 2001 (The International Human Genome Sequencing Consortium, 2001; Venter et al., 2001), sequencing technology has become approximately one million times faster and cheaper, making it possible for individual labs to generate as much sequence data as the entire Human Genome Project in just a few days. In the context of metagenomics experiments, this makes it possible to sample a complex mixture of microbes by “shotgun” sequencing, which involves simply isolating DNA, preparing the DNA for sequencing, and sequencing the mixture as deeply as possible. Shotgun sequencing is relatively unbiased compared to targeted sequencing methods (Venter et al., 2004), including widely-used 16S ribosomal RNA sequencing, and it has the additional advantage that it captures any species with a DNA-based genome, including eukaryotes that lack a 16S rRNA gene. Because it is unbiased, shotgun sequencing can also be used to estimate the abundance of each taxon (species, genus, phylum, etc.) in the original sample, by counting the number of reads belonging to each taxon.

Along with the technological advances, the number of finished and draft genomes has also grown exponentially over the past decade. At present there are thousands of complete bacterial genomes, 20,000 draft bacterial genomes, and 80,000 full or partial virus genomes in the public GenBank archive (Benson et al., 2015). This rich resource of sequenced genomes now makes it possible to sequence uncultured, unprocessed microbial DNA from almost any environment, ranging from soil to the deep ocean to the human body, and use computational sequence comparisons to identify many of the formerly hidden species in these environments (Riesenfeld, Schloss & Handelsman, 2004). Several accurate methods have appeared that can align a sequence “read” to a database of microbial genomes rapidly and accurately (see below), but this step alone is not sufficient to estimate how much of a species is present. Complications arise when closely related species are present in the same sample–a situation that arises quite frequently–because many reads align equally well to more than one species. This requires a separate abundance estimation algorithm to resolve. In this paper, we describe a new method, Bracken, that goes beyond simply classifying individual reads and computes the abundance of species, genera, or other taxonomic categories from the DNA sequences collected in a metagenomics experiment.

When it was first published in 2014, the Kraken metagenomics classifier represented a major enhancement in the speed with which large metagenomics sequence data could be processed (Wood & Salzberg, 2014), running over 900 times faster than MegaBlast (Morgulis et al., 2008), the closest competitor at the time. Kraken’s success and accuracy rely on its use of a very large, efficient index of short sequences of length k, which it builds into a specialized database. If k is chosen appropriately, then most sequences of length k in the database will be unique to a single species, and many will also be unique to a particular strain or genome. Larger values of k will yield a database in which even more of each genome is uniquely covered by k-mers; obviously, though, k should not be longer than the length of a sequencing read, and metagenomics projects currently generate reads as short as 75–100 base pairs (bp). Longer k-mers are also more likely to contain errors, meaning that more reads will be left unclassified if k is too long. Smaller k-mers, in contrast, will yield higher sensitivity because the minimum match length is shorter.

When used to identify the taxonomic label of metagenomics sequences, the Kraken system for classification of metagenomics sequences is extremely fast and accurate (Wood & Salzberg, 2014). When classifying raw sequence reads, though, many reads correspond to identical regions between two or more genomes. (The number of such ambiguous reads decreases as reads get longer.) Kraken solves this problem by labeling the sequence with the lowest common ancestor (LCA) of all species that share that sequence, as discussed further below.

### Ambiguity among microbial species and strains

As the database of bacterial genomes has grown, an increasing number of genomes share large portions of their sequence with other genomes. In many cases, these genomes are nearly identical; indeed, sequencing has revealed to scientists that many formerly distinct species and genera are far closer than were known prior to sequencing. Many species have been renamed as a result, in a process that is continual and ongoing, but many other species have retained their old names, often for historical or other reasons.

For example, the species *Mycobacterium bovis* is over 99.95% identical to *Mycobacterium tuberculosis* (Garnier et al., 2003), and many cases of human tuberculosis are caused by *M. bovis* (which also infects cows) rather than *M. tuberculosis* (Grange, 2001). Their high sequence identity indicates that they should be considered as two strains of a single species, but they retain different species names. As a compromise, taxonomists have created the category *Mycobacterium tuberculosis complex* (Brosch et al., 2002) to represent a collection of taxa that now includes more than 100 strains of five different species. This category sits above the species level but below the genus level in the current microbial taxonomy, but it can best be described as a species.

Other examples are numerous and still growing. The three species *Bacillus anthracis* (the causative agent of anthrax), *Bacillus cereus*, and *Bacillus thuringiensis* are well over 99% identical and should all be designated as a single species (Helgason et al., 2000), although their names have not been changed despite their near-identity revealed by sequencing. As a compromise, taxonomists created the category *Bacillus cereus group*, between the level of species and genus, to include these three species and at least five others (Liu et al., 2015), all of which are extremely similar to one another. In some cases, two organisms that should be called the same species may even have different genus names. For example, *Escherichia coli* and *Shigella flexneri* are classified in different genera, but we know from sequence analysis that they represent the same species (Lan & Reeves, 2002).

Failure to recognize the mutability of the bacterial taxonomy can lead to erroneous conclusions about the performance of metagenomic classifiers. For example, one recent study (Peabody et al., 2015) created a mock community of 11 species, one of which was *Anabaena variabilis* ATCC 29413, not realizing that this genome had been renamed and was synonymous with species in the genus *Nostoc* (Thiel et al., 2014). When *Anabaena* was removed from the database, Kraken correctly identified the reads as *Nostoc*, but Peabody et al. erroneously considered all these reads to be misclassified.

### Classification versus abundance estimation

Kraken attempts to assign a taxonomy label to every read in a metagenomics sample using a custom-built database that may contain any species the user chooses. Among the current set of finished bacterial and archaeal genomes, hundreds of species can be found for which large fractions of their sequence are identical to other genomes belonging to distinct strains, species, or even genera. The reads arising from common regions in these species result in a tie when analyzed with Kraken’s classification algorithm, so Kraken correctly reports only the lowest common ancestor (LCA) (Wood & Salzberg, 2014). It follows that for well-populated clades with low genome diversity, Kraken only reports species-level assignments for reads from unique regions, and a true indication of total abundance can only be made by taking both species and genus (or higher) level assignments into account. This implies that for some species, the majority of reads might be classified at a higher level of the taxonomy. Kraken thus leaves many reads “stranded” above the species level, meaning that the number of reads classified directly to a species may be far lower than the actual number present.

Therefore, any assumption that Kraken’s raw read assignments can be directly translated into species- or strain-level abundance estimates (e.g., Schaeffer et al., 2015) is flawed, as ignoring reads at higher levels of the taxonomy will grossly underestimate some species, and creates the erroneous impression that Kraken’s assignments themselves were incorrect.

Nonetheless, metagenomics analysis often involves estimating the abundance of the species in a particular sample. Although we cannot unambiguously assign each read to a species, we would like to estimate how much of each species is present, specifically by estimating the number or percentage of reads in the sample. Several software tools have been developed to estimate species abundances in metagenomics samples [MetaPhlAn, ConStrains, GAAS, GASiC, TAEC, GRAMMy] (Angly et al., 2009; Lindner & Renard, 2012; Luo et al., 2015; Segata et al., 2012; Sohn et al., 2014; Xia et al., 2011). These tools, however, employ different strategies for read-level classification which are not always as accurate and efficient as Kraken’s k-mer approach (Lindgreen, Adair & Gardner, 2016). Rather than re-engineer Kraken to address the ambiguous read classification issue and to provide abundance estimates directly, we decided to implement the new species-level abundance estimation method described here as a separate program. This preserves both backwards compatibility for existing Kraken users, and offers the ability to generate more accurate species abundance estimates for datasets already processed by Kraken. Note that if Kraken fails to identify a species (e.g., if the species was missing from the Kraken database), Bracken too will not identify that species.

## MATERIALS AND METHODS

**Figure 1:**
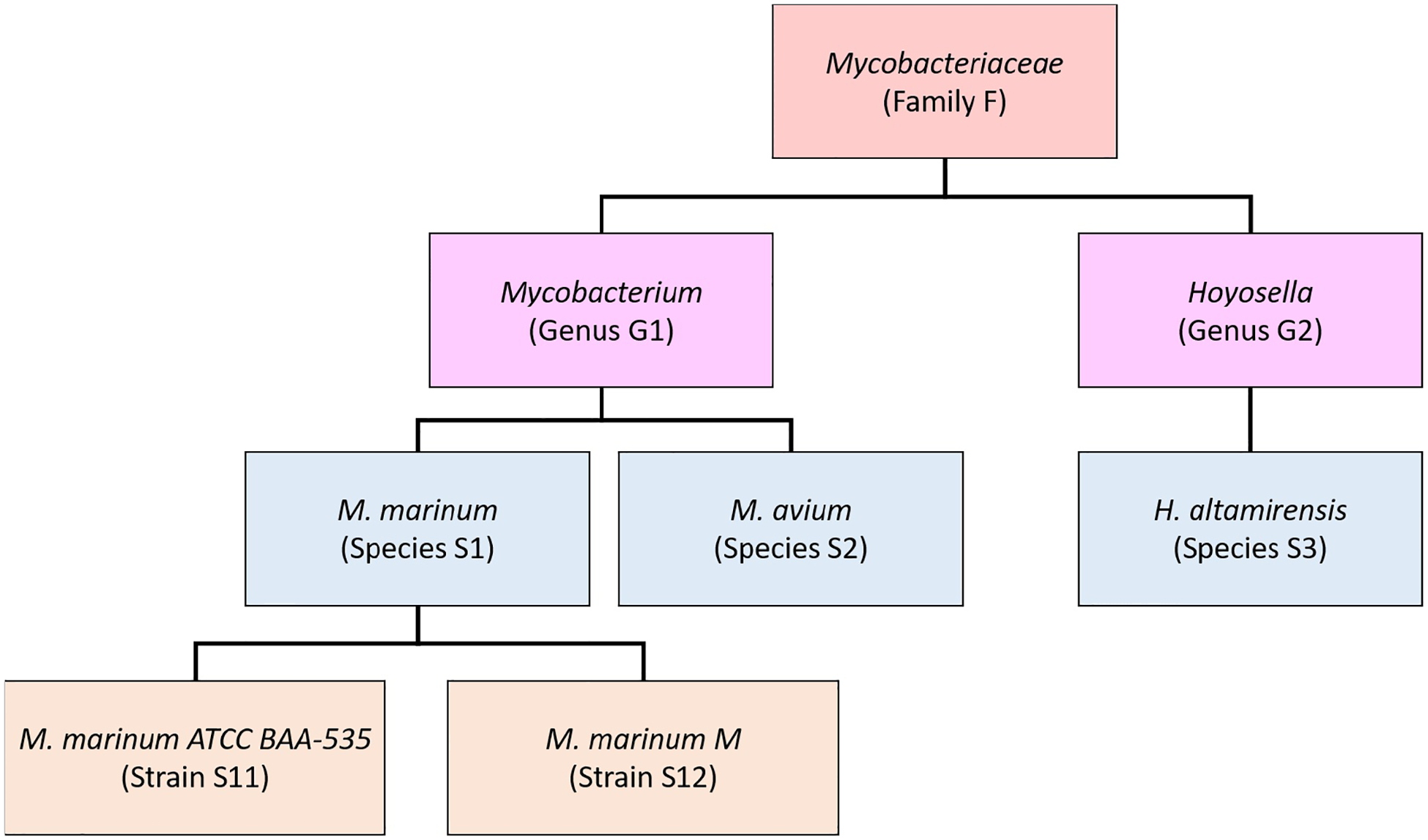
Schematic showing a partial taxonomic tree for the *Mycobacteriaceae* family.

Our new method, Bracken (Bayesian Reestimation of Abundance after Classification with KrakEN), estimates species abundances in metagenomics samples by probabilistically re-distributing reads in the taxonomic tree. Reads assigned to nodes above the species level are distributed down to the species nodes, while reads assigned at the strain level are re-distributed upward to their parent species. For example, in Fig. 1 we would distribute reads assigned to the *Mycobacteriaceae* family and the *Mycobacterium* genus down to *M. marinum* and *M. avium*, and reads assigned to each *M. marinum* strain would be reassigned to the *M. marinum* species. As we show below, Bracken can easily reestimate abundances at other taxonomic levels (e.g., genus or phylum) using the same algorithm.

In order to re-assign reads classified at higher-level nodes in the taxonomy, we need to compute a probabilistic estimate of the number of reads that should be distributed to the species below that node. To illustrate using the nodes in Fig. 1, we need to allocate all reads assigned to *Mycobacterium* (G1) to *M. marinum* (S1) and *M. avium* (S2) below it, and reads assigned to the *Mycobacteriaceae* would have to be allocated to *M. marinum* (S1), *M. avium* (S2), and *Hoyosella altamirensis* (S3).

Reallocating reads from a genus-level node in the taxonomy to each genome below it can be accomplished using Bayes’ theorem, if the appropriate probabilities can be computed. Let $P(S_{i})$ be the probability that a read in the sample belongs to genome $S_{i}$, $P(G_{j})$ be the probability that a read is classified by Kraken at the genus level $G_{j}$, and $P(G_{j}|S_{i})$ be the probability that a read from genome $S_{i}$ is classified by Kraken as the parent genus $G_{j}$. Then the probability that a read classified at genus $G_{j}$ belongs to the genome $S_{i}$ can be expressed as Eq. (1):

(1) $$P(S_{i}|G_{j})=\frac{P(G_{j}|S_{i})P(S_{i})}{P(G_{j})}.$$

Note that because we began by assuming that a read was classified at node $G_{j}$, $P(G_{j})=1$.

Next we consider how to compute $P(G_{j}|S_{i})$, the probability that a read from genome $S_{i}$ will be classified by Kraken at the parent genus $G_{j}$. We estimate this probability for reads of length r by classifying the sequences (genomes) that we used to build the database using that same database, as follows. For each k-mer in the sequences, Kraken assigns it a taxonomy ID by a fast lookup in its database. To assign a taxonomy ID for a read of length r, Kraken examines all k-mer classifications in that read. For example, for k=31 and r=75, the read will contain 45 k-mers. Our procedure examines, for each genome in the database, a sliding window of length r across the entire genome.

To find the taxonomy ID Kraken would assign to each window, we simply find the deepest taxonomy node in the set of k-mers in that window. Since each k-mer in a database sequence is assigned to a taxonomy ID somewhere along the path from the genome’s taxonomy ID to the root, the highest-weighted root-to-leaf path (and thus the Kraken classification) corresponds to the deepest node.

For each genome $S_{i}$ of length $L_{i}$ we thus generate ($L_{i}-r+1$) mappings to taxonomical IDs. For node $G_{j}$, we then count the number of reads from ${\text{S}}_{i}$ that are assigned to it, $N_{G_{j}(S_{i})}$. $P(G_{j}|S_{i})$ is then the proportion of reads from $S_{i}$ that were assigned to the genus node $G_{j}$; i.e., $P(G_{j}|S_{i})=N_{G_{j}(S_{i})}/L_{i}-r+1$. We also calculate the proportion of reads from $S_{i}$ that were assigned to every node from genome $S_{i}$ to the root node of the taxonomy tree.

The final term that we must calculate from Eq. (1) is $P(S_{i})$, the probability that a read in the sample belongs to genome $S_{i}$, which is computed in relation to other genomes from the same genus. For example, if the sample contains three genomes in the same genus, and if 30% of all reads from those three genomes belong to $S_{i}$, then $P(S_{i})=0.3$. We estimate this probability using the reads that are uniquely assigned by Kraken to genome $S_{i}$, as follows.

If we let $U_{Si}$ be the proportion of genome $S_{i}$ that is unique, then

(2) $$U_{S_{i}}=\frac{N_{S_{i}}}{L_{i}-r+1}$$

where $N_{si}$ is the number of k-mers of length r that are uniquely assigned to genome $S_{i}$ by Kraken, and $L_{i}$ is the genome length. For example, if $L_{i}=1$ Mbp and only 250,000 k-mers are unique to genome $S_{i}$, then $U_{Si}=0.25$.

Then, using the number of reads $K_{Si}$ from a sample that Kraken actually assigns to $S_{i}$, we can estimate the number of reads that likely derive from $S_{i}$ as:

(3) $${\hat{K}}_{S_{i}}=\frac{K_{S_{i}}}{U_{S_{i}}}.$$

For example, if Kraken classifies 1,000 reads as genome $S_{i}$ and 25% of the reads from $S_{i}$ are unique, then we would estimate that 4,000 reads (1,000/0.25) from $S_{i}$ are contained in the sample.

If genus $G_{j}$ contains n genomes, we estimate the number of reads ${\hat{K}}_{S}$ for each of the n genomes and then calculate $P(S_{i})$ by:

(4) $$P(S_{i})=\frac{{\hat{K}}_{S_{i}}}{\sum_{a=1}^{n}{\hat{K}}_{S_{a}}}.$$

Using this result in Eq. (1) above allows us to compute $P(S_{i}|G_{j})$ for each genome $S_{i}$. Each probability $P(S_{i}|G_{j})$ is then used to estimate the proportion of the reads assigned to genus $G_{j}$ that belong to each of the genomes below it.

These calculations are repeated for each taxonomic level above the genus level (family, class, etc.), with read distribution at each level going to all genomes classified within that taxonomic subtree.

To compute species abundance, any genome-level (strain-level) reads are simply added together at the species level. In cases where only one genome from a given species is detected by Kraken in the dataset, we simply add the reads distributed downward from the genus level (and above) to the reads already assigned by Kraken to the species level. In cases where multiple genomes exist for a given species, the reads distributed to each genome are combined and added to the Kraken-assigned species level reads. The added reads give the final species-level abundance estimates.

This method can also estimate abundance for other taxonomic levels. In such cases, only higher nodes within the taxonomy tree undergo read distribution. After distributing reads downward, we estimate abundance for a node at the level specified by combining the distributed reads across all genomes within that node’s subtree.

### Software and data availability

Bracken is written in Perl and Python and is freely available for download at http://ccb.jhu.edu/software/bracken/. The reads from the skin microbiome experiment are freely available from NCBI under BioProject PRJNA316735.

## RESULTS AND DISCUSSION

We applied the statistical re-assignment method described here to create species-level abundance estimates for several metagenomics data sets. The overall procedure works as follows. First, we compute a set of probabilities from the Kraken database by computing, for every sequence of length R in every genome, where it will be assigned in the taxonomy (see ‘Methods’). For our experiments, we set R=75 as our datasets contain 75-bp reads. Bracken can use these probabilities for any metagenomics data set, including data with different read lengths, although the estimates might be slightly improved by re-computing with a read length that matches the experimental data.

Second, we run Kraken on the dataset to produce read-level taxonomic classifications. We then apply our abundance estimator, Bracken, which uses the numbers of reads assigned by Kraken at every level of the taxonomy to estimate the abundances at a single level (e.g., species). Note that to exclude false positives, Bracken ignores species with counts below a user-adjustable threshold. In our experiments, we selected a threshold of 10 reads.

### Experiments on a 100-genome metagenomics data set

For our first experiments, we used a data set containing simulated Illumina reads from 100 genomes. This data, which we call here the i100 dataset, was used previously in a comparison of metagenomic assembly algorithms (Mende et al., 2012). The data contains 53.3 million paired reads (26.7M pairs) from 100 genomes representing 85 species. The reads have error profiles based on quality values found in real Illumina reads (Mende et al., 2012). The i100 dataset includes several very challenging genomes for this task, including multiple strains and species in the genera *Bacillus* and *Mycobacteria*, some of which are nearly identical to one another. The i100 data are freely available at http://www.bork.embl.de/~mende/simulated_data.

**Figure 2:**
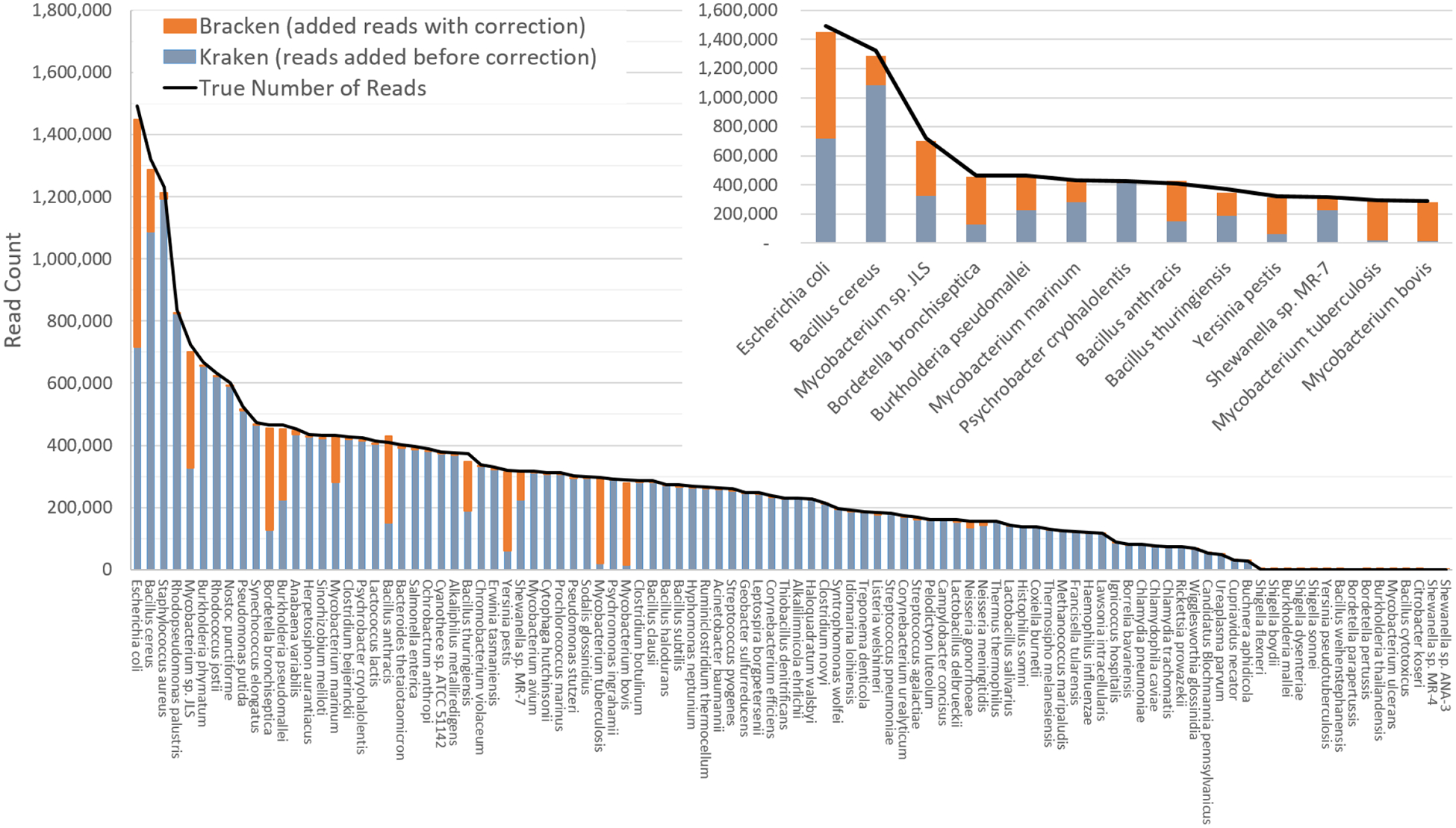
Estimates of species abundance in the i100 metagenomics dataset computed by Kraken (blue) and Bracken (blue + orange). For this result, the Kraken database contained 693 genomes that included the i100 genomes. The smaller graph displays results for the subset of species for which Bracken made the largest adjustments. The black line shows the true number of reads from each species. Precise numbers for the Kraken classification, true read counts, and Bracken estimates are contained in Table S2A.

The difficulty of estimating species abundance increases as the database itself contains more species. For example, it would clearly be easier to estimate abundances in the i100 dataset if we used a Kraken database containing only the 100 genomes in that dataset. To make the problem more realistic, we built two different databases and estimated abundance using both. The first (“small”) database contains 693 genomes including the i100 genomes; this is the full database from the simulation study by Mende et al. (2012). The results when using the small database for classification are shown in Fig. 2. For several species, the initial Kraken numbers (reads assigned to a particular species) are far too low, because many of the reads (for some genomes, a large majority) were assigned labels at the genus level or above. After reestimation with Bracken, these reads were redistributed to the species level, with the result that almost all the abundance estimates were 98–99% correct, as shown in the figure.

The second (“large”) database contains all genomes used in the synthetic and spike-in experiments, as well as a broad background of bacterial genomes. In particular, it includes all complete bacterial and archaeal genomes from RefSeq as of 25 July 2014 (archived at ftp://ftp.ncbi.nlm.nih.gov/genomes/archive/old_refseq), which total 2596 distinct taxa, plus those i100 genomes that were not present in the RefSeq data. (We excluded draft genomes because they often contain vector sequences or other contaminants.) We also added the nine genomes used in our skin bacteria spike-in experiment (described below) resulting in a total of 2635 distinct taxa. The complete list of sequences in the large database can be found in Table S1. The resulting Kraken database has a size of 74 GB.

**Figure 3:**
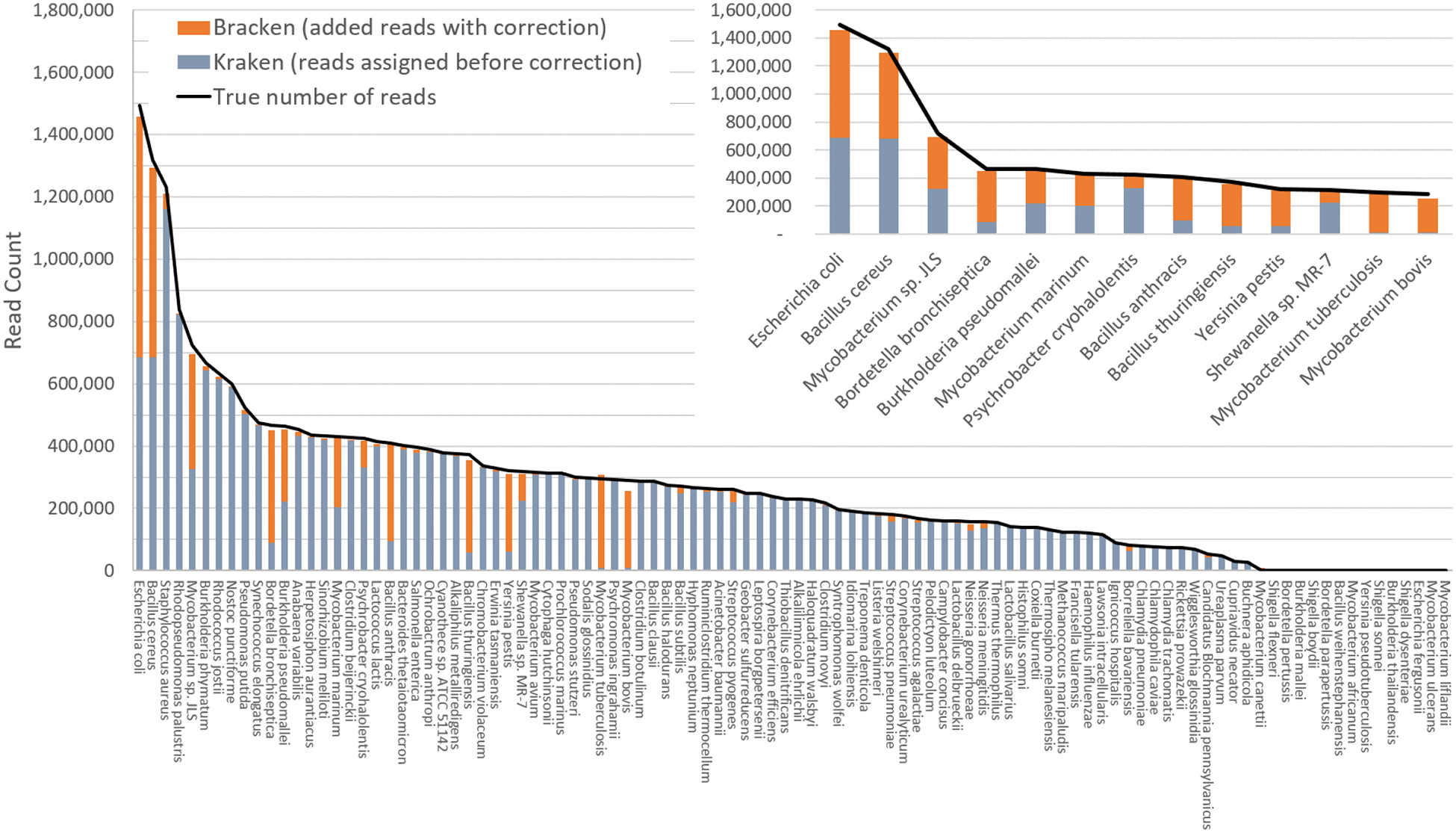
Estimates of species abundance computed by Kraken (blue) and Bracken (blue + orange) for the i100 metagenomics data. For this result, the Kraken database contained 2,635 distinct bacterial and archaeal taxa. The black line shows the true number of reads from each species. The smaller graph displays results for the subset of species for which Bracken made the largest adjustments. Precise numbers for the Kraken classification, true read counts, and Bracken estimates are contained in Table S2B.

Figure 3 shows results when using the large database to estimate abundance for the i100 genomes. This test is much more difficult because of the large number of similar and near-identical genomes in the database. Many more reads are ambiguous, mapping identically to two or more species, which means that Kraken assigns them to the LCA of those species. Nonetheless, Bracken brings the estimated abundance of all species within 4% of the true abundance, and most fall within 1%. Note that when the re-estimation procedure distributes reads from higher nodes in the taxonomy down to multiple species within a single genus, it may over-estimate one species and underestimate its sister species if the re-allocation is imperfect.

Tables S2A–S2B contains the detailed numbers for all species in Figs. 2 and 3, along with an error rate for each species in the i100 data, expressed as the difference between the true and estimated proportions. We calculated the average error as:

(5) $$\frac{1}{n}\overset{n}{\underset{i=1}{\sum }}\frac{\left|R_{\text{true}}^{\left(i\right)}-R_{\text{est}}^{\left(i\right)}\right|}{R_{\text{true}}^{\left(i\right)}}$$

where n is the number of species in the i100 data, $R_{\text{true}}^{\left(i\right)}$ is the true number of reads for species i, and $R_{\text{est}}^{\left(i\right)}$ is the Bracken estimate of the number of reads for species i. When using the small database, the average relative error of Bracken is 1.75% across all 85 species in the i100 data. For the larger database, the average relative error is 1.89%. We also calculated false positive rates for the i100 data as the percentage of total reads incorrectly classified after Bracken abundance estimation. For the small database, the false positive rate is 0.13% and for the large database, the false positive rate is 0.24%.

**Figure 4:**
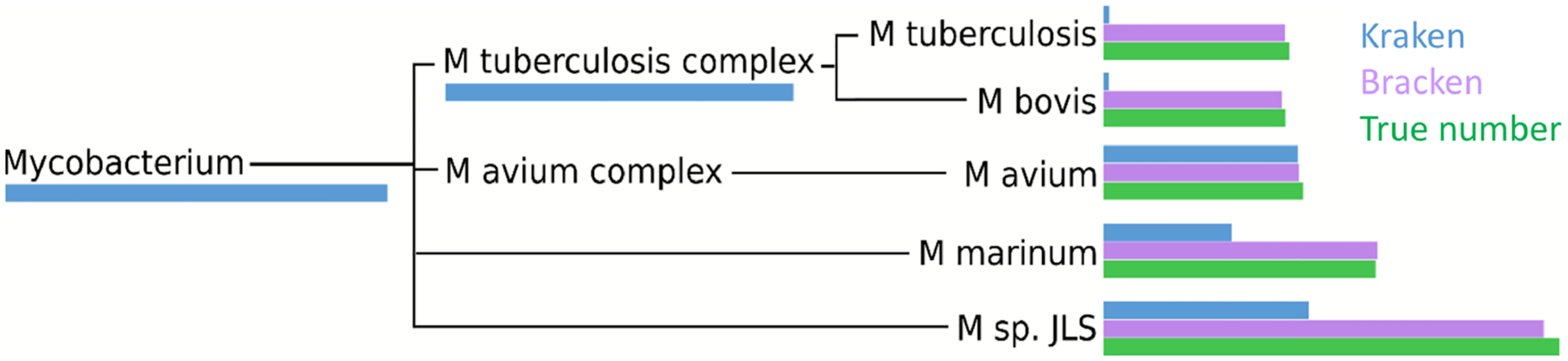
Number of reads within the *Mycobacterium* genus as assigned by Kraken (blue), estimated by Bracken (purple) and compared to the true read counts (green). Initially, Kraken assigned only 325,073 reads to *Mycobacterium sp. JLS* although 722,880 reads originated from this species. Bracken reassigned 370,601 reads from the *Mycobacterium* genus to *M. sp. JLS*. Bracken’s re-estimated abundance for *M. sp. JLS* is much closer to the true read count. Table S3 contains precise numbers for all species shown here.

Within the i100 genomes, the five species belonging to the *Mycobacterium* genus (*M. tuberculosis, M. bovis, M. avium, M. marinum*, and *M. sp. JLS*) pose a particular challenge for abundance estimation due to the similarities among their individual genomes. For example, Kraken classified only 9,733 *M. tuberculosis* reads at the species level, and classified the remaining 285,414 reads as either *Mycobacterium* (a genus) or *M. tuberculosis complex* (a taxonomic class intermediate between genus and species), as shown in Fig. 4 and Table S3. For these *Mycobacteria* genomes, Bracken reallocated the reads from higher-level nodes to yield species abundance estimates within 4% of the true abundance. Figure 4 and Table S3 show the number of reads assigned to each species by Kraken, the true number of reads, and the number of reads assigned to each species by Bracken after abundance reestimation.

The five species of the *Mycobacterium* genus also provide an example of potential overestimation by Bracken. Bracken apportions all ambiguous reads classified by Kraken at the genus level (and above) to the existing species identified by Kraken. Because Bracken uses a probabilistic method in distributing the reads, one species may receive too many reads while another may receive too few. For example, Kraken assigned 543,916 reads to *M. tuberculosis complex*. Bracken re-allocated 296,543 of these reads to *M. tuberculosis* and the remaining 247,453 reads to *M. bovis*. When added to Kraken’s original assignments, Bracken estimated that 306,792 reads belonged to *M. tuberculosis* (11,645 reads more than the true number) that 256,927 reads belonged to *M. bovis* (31,473 reads less than the true number). It is likely that some of the additional reads Bracken allocated to *M. tuberculosis* originated from *M. bovis* instead. However, despite the over- and under-estimation, Bracken’s estimates fell within 4% of the true number of reads for both species.

If *M. bovis* were excluded from the database, the 8,965 reads unique to *M. bovis*, as identified by Kraken, would be unclassified, while all 543,916 reads assigned to the *M. tuberculosis complex* would assigned to *M. tuberculosis* by Kraken. These reads would no longer be ambiguous because no other *Mycobacterium* species from the *M. tuberculosis complex* would be present in the database. In general, reads belonging to species excluded from the database will either be assigned to species with very high similarity to the missing species or will remain unclassified.

### Experiments on a real metagenomics sample created from known species

For a more realistic evaluation of the performance of Bracken, we generated new sequence data using a set of bacteria that are commonly found on healthy human skin. This mock community was assembled by combining purified DNA from nine isolates that were identified and sequenced during the initial phase of the Human Microbiome Project (Human Microbiome Project, 2012): *Acinetobacter radioresistens* strain SK82, *Corynebacterium amycolatum* strain SK46, *Micrococcus luteus* strain SK58, *Rhodococcus erythropolis* strain SK121, *Staphylococcus capitis* strain SK14, *Staphylococcus epidermidis* strain SK135, *Staphylococcus hominis* strain SK119, *Staphylococcus warneri* strain SK66, and *Propionibacterium acnes* strain SK137. To generate the skin microbiome community, purified DNA was obtained from the Biodefense and Emerging Infections Research Resources Repository (BEI Resources). Each of the nine bacterial isolates was grown under conditions recommended by BEI Resources, collected by centrifugation during log growth phase at a 600nm optical density (OD^600^) of 0.8–1.2, and genomic DNA was isolated using MasterPure DNA isolation reagents (Epicentre). Purified genomic DNA was quantified using the high sensitivity picogreen assay (Invitrogen), pooled in equal amounts by mass, and prepared for sequencing using Nextera XT library preparation reagents (Illumina). The sample was then sequenced on a HiSeq sequencer, generating a total of 78,439,985 million read pairs (157 million reads), all of them 100 bp in length. These were then classified as pairs by Kraken, which concatenates the two reads from each pair and assigns them to a single taxonomic category.

**Figure 5:**
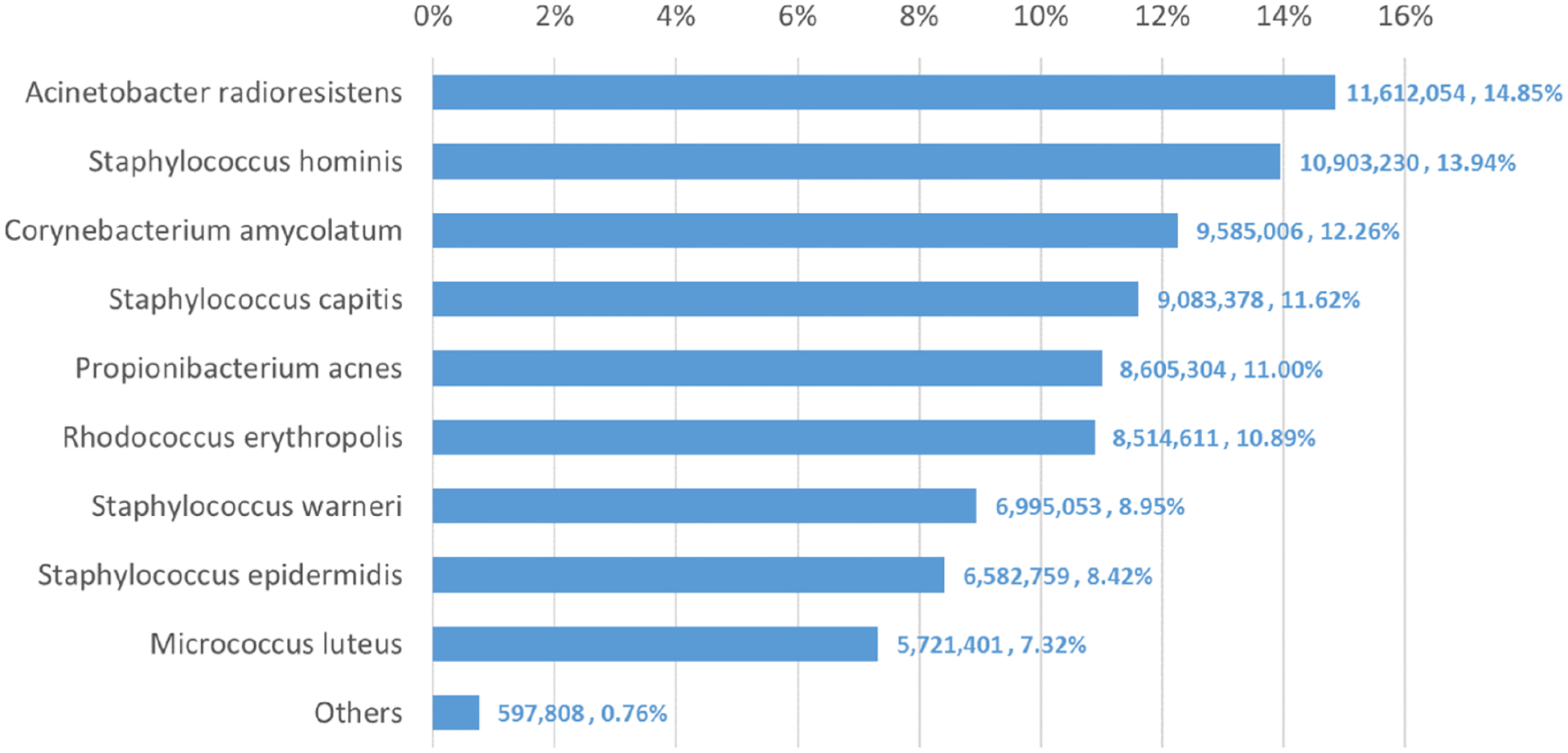
Estimates of species abundance made by Bracken for the metagenomics community containing isolates of nine bacterial species commonly found on human skin. Precise numbers can be found in Table S4.

We used Bracken to estimate both species and genus-level abundance in the skin microbiome community. In the Bracken results, the nine true species comprise over 99% of the species-level abundance estimates. The mixture was created with approximately equal amounts of each of the nine genomes, so the expectation was that each species would account for ~11% of the total. However, as shown in Fig. 5, the estimates varied from 7.3% to 14.8%. Details for the exact number of reads assigned by Kraken and the abundance estimates by Bracken are shown in Table S4.

Deviations from the expected abundance could arise from a variety of factors. The process of quantifying DNA and mixing in equal amounts can be influenced by pipetting consistency. Second, library amplification by PCR, an integral step in the Nextera library preparation process, can exaggerate small differences in quantities and lead to significant biases in abundance (Bowers et al., 2015). We examined a sample of the classified reads by hand, and could find no evidence that Kraken mis-classified reads from *M. luteus* (the smallest portion of the community, estimated at 7.3%) to any of the other species or genera. The abundances found in this data, therefore, may correspond fairly closely with the true abundances.

The genus-level abundance estimates computed by Bracken also correspond closely to the expected abundances for the six genera included in the sample. Four of the nine species belong to the genus Staphylococcus, which was thus expected to comprise 44% (4 × 11%) of the sample. The Bracken estimate was 43.3%. Each of the other genus classifications has only one species present, and their abundance estimates are the same for both genus and species.

The comparison between the Kraken classification of reads and Bracken’s reassignment revealed that the nine species are sufficiently distinct to allow Kraken to classify a large majority of reads at the species level, with very few reads being classified at higher levels of the taxonomy. Specifically, Kraken classified 76.4 million reads to the nine species included in the sample. Only 1.3 million reads out of the 78.2 million total (1.6%) were classified by Kraken at the genus level or above. (The remaining reads were unclassified.) In this case Bracken does not provide a substantial benefit, because reassignment of the 1.3 million reads could yield at most a 1.6% change in the estimated composition of the sample.

### Abundance estimation timing and resource requirements

In the i100 data experiment with the small database, we used 188 gigabytes (GB) of RAM with 10 threads to build the Kraken database and generate the k-mer distribution file required by Bracken. In total, these steps completed in about 2 h and yielded files that can be used across multiple datasets. The resulting Kraken database and distribution files use 53 GB of space. Kraken classification of the i100 dataset took 18 min, using 10 threads and 107 GB of RAM. This step is limited by the size of the database, which is loaded into RAM during classification. Bracken alone runs in under a second, using 35 MB of RAM. The Kraken classification file for the i100 data is 1.6 GB, while Bracken abundance estimation files require ~65 KB of space. Table S5 lists detailed timing, RAM, and space requirements for each file and step of the Bracken abundance estimation algorithm.

## CONCLUSION

Estimating the abundance of species, genera, phyla, or other taxonomic groups is a central step in the analysis of many metagenomics datasets. Metagenomics classifiers like Kraken provide a very fast and accurate way to label individual reads, and at higher taxonomic levels such as phyla, these assignments can be directly translated to abundance estimates. However, many reads cannot be unambiguously assigned to a single strain or species, for at least two reasons. First, many bacterial species are nearly identical, meaning that a read can match identically to two or more distinct species. Second, the bacterial taxonomy itself is undergoing constant revisions and updates, as genome sequencing reveals the need to re-assign species to new names. These revisions sometimes create new taxa that share near-identical sequence with a distinct species. In these situations, Kraken correctly assigns the read to a higher-level taxonomic category such as genus or family. This creates a problem in that Kraken’s classifications cannot be used directly for species abundance estimation.

Bracken addresses this problem by probabilistically re-assigning reads from intermediate taxonomic nodes to the species level or above. As we have shown here, these re-assignments produce species-level abundance estimates that are very accurate, typically 98% correct or higher. For genus-level abundance, accuracy is even higher because fewer reads have ambiguous assignments at that level. For abundance estimation at higher levels, ranging from family up to phylum, Kraken’s original read assignments can be used directly to create abundance estimates.

## Supplementary Material

Supplementary Table 1

Supplementary Tables 2A-B

Supplementary Table 3

Supplementary Table 4

Supplementary Table 5

## Data Availability

The following information was supplied regarding data availability: Bracken is written in Perl and Python and is freely available for download at http://ccb.jhu.edu/software/bracken/. The reads from the skin microbiome experiment are freely available from NCBI under BioProject PRJNA316735.
